# Analysis of long-term effect of ureteral balloon dilatation combined with internal and external drainage tube in the treatment of benign ureteral stricture

**DOI:** 10.1186/s12894-022-00952-6

**Published:** 2022-01-13

**Authors:** Haohao Lu, Chuansheng Zheng, Bin Liang, Bin Xiong

**Affiliations:** 1 Interventional Therapy Department, Wuhan Union Hospital, Tongji Medical College, Huazhong University of Science and Technology, Jiefang Avenue #1277, Wuhan, 430022 China; 2 Hubei Province Key Laboratory of Molecular Imaging, Wuhan, 430022 China

**Keywords:** Ureteral stricture, Balloon dilatation, Drainage, Interventional therapy, Minimally invasive treatment, Hydronephrosis

## Abstract

**Purpose:**

There are few reports about balloon dilatation combined with internal and external drainage tube in the treatment of ureteral stricture under interventional therapy. The aim of the study is to explore the safety, effectiveness and long-term efficacy of this treatment strategy.

**Materials and methods:**

It is a retrospective and observational study. From October 2013 to October 2016, 42 patients with benign lower ureteral stricture received interventional treatment. Balloon dilatation combined with internal and external drainage tube implantation were used. There were 25 male patients and 17 female patients. There were 7 cases (16.7%) with congenital ureteral stricture, 12 cases (28.6%) with inflammation, 15 cases (35.7%) with ureteral stricture after lithotomy or lithotripsy, and 8 cases (19.0%) with ureteral stricture after pelvic or abdominal surgery. After the drainage tube was removed, B ultrasound, enhanced CTU or IVP of urinary system were reexamined every six months. The follow-up time was 12–60 months.

**Results:**

The age was 52.9 ± 11.6 years. The length of ureteral stricture was 1.1 ± 0.5 cm. 42 patients completed interventional treatment, the technical success rate was 100%, no ureteral perforation, rupture or other complications were identified. Preoperative urea nitrogen 9.2 ± 2.3 mmol/L and creatinine 175.8 ± 82.8umol/L. Urea nitrogen and creatinine were 3.8–9.1 mmol/L and 45.2–189.6 umol/L when removing the drainage tube. There were significant differences in the levels of urea nitrogen and creatinine before and after tube removal (*P* < 0.05). The ureteral patency rate was 100% at 6 months, 93% at 12 months, 83% at 18 months, 79% at 24 months, 76% at 30 months and 73% at 36–60 months. The overall success rate was 73%. Multivariate Cox regression analysis showed that stenosis length was a risk factor for postoperative patency (*P* < 0.05).

**Conclusion:**

Balloon dilatation combined with internal and external drainage tube implantation in the treatment of benign lower ureteral stricture is safe and effective.

## Introduction

Ureteral stricture is a relatively rare urinary system disease, which refers to ureteral stenosis caused by various reasons, eventually leading to hydronephrosis or renal failure [1]. There are many causes of ureteral stricture, including congenital and secondary ureteral stricture. The causes of secondary ureteral stricture include calculus, inflammation, injury after lithotripsy [2], ureteral related operation, tumor invasion, retroperitoneal fibrosis, iatrogenic injury caused by abdominal and pelvic surgery, radiotherapy, etc. [3, 4]. Especially iatrogenic injury, although with the improvement of endoscopic technology and surgical methods, the incidence of iatrogenic injury decreased continuously, from 10% 20 years ago to 3–6% 10 years ago, and even less than 0.2% reported in recent years [5, 6]. However, due to the increase of the number of operations, the absolute number of iatrogenic injuries is rising, which should be paid great attention by medical staff. Commonly used treatments for ureteral strictures include open surgery and minimally invasive treatment. Internal and external drainage tubes are mostly used for drainage of the biliary system [7]. There were few reports of balloon dilatation combined with internal and external drainage tube in the treatment of ureteral stricture under interventional therapy, and the observation of long-term therapeutic effect of balloon dilatation is scarce in the literature. This study retrospectively collected the data of patients with ureteral stricture treated by balloon dilatation combined with internal and external drainage tube in the Interventional Therapy Department of our hospital, to analyse the safety, effectiveness and long-term efficacy of this treatment method.

## Materials and methods

### General information

From October 2013 to October 2016, clinical data from 42 patients with benign lower ureteral stenosis were collected, who were treated in the Interventional Therapy Department of Wuhan Union Hospital Affiliated to Tongji College of Huazhong University of science and technology. Balloon dilatation combined with internal and external drainage tube implantation were used. The study was approved by the hospital ethics committee, and all patients' data collection was informed and agreed by the patients and their families. All patients had unilateral single segment benign lower ureteral stenosis. B-ultrasound, enhanced CTU or/and MRU examination were performed before the operation to determine the cause, location and length of the stenosis. Antegrade pyelography was performed during the operation. All patients had different degrees of hydronephrosis, which were divided into mild (less than 3 cm), moderate (3–4 cm) and severe (more than 4 cm).

Intraoperative consumables: percutaneous renal puncture Kit (NPAS-100-RH-NT, Cook, USA), internal and external drainage catheter (ULT10.2-38-40-P-32S-CLB-RH, Cook, USA), 6F vascular sheath (TERUMO6F-10CM, Terumo, Japan), 0.035 inch guide wire (RF*GA35153M, RF*PA35263M, Terumo, Japan), 6 mm/8 mm diameter balloon (PTA5-35-135-6-8.0, PTA5-35-135-8-8.0, Cook, USA), 5F multipurpose catheter (MPA1, Cordis, USA).

### Method

**Fig. 1:**
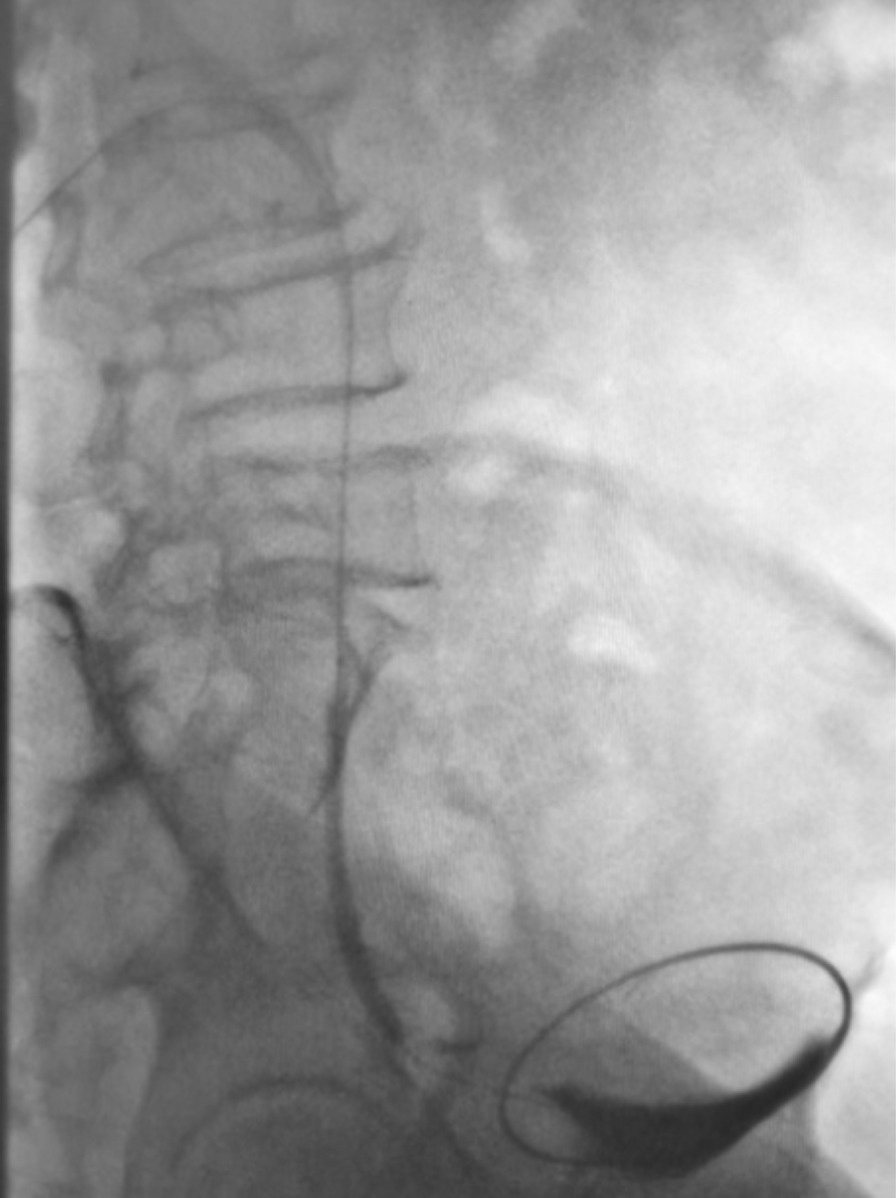
Fluoroscopic image showing the guidewire inserted through a percutaneous access to the urinary bladder, ureteral stenosis was dilated with a balloon

**Fig. 2:**
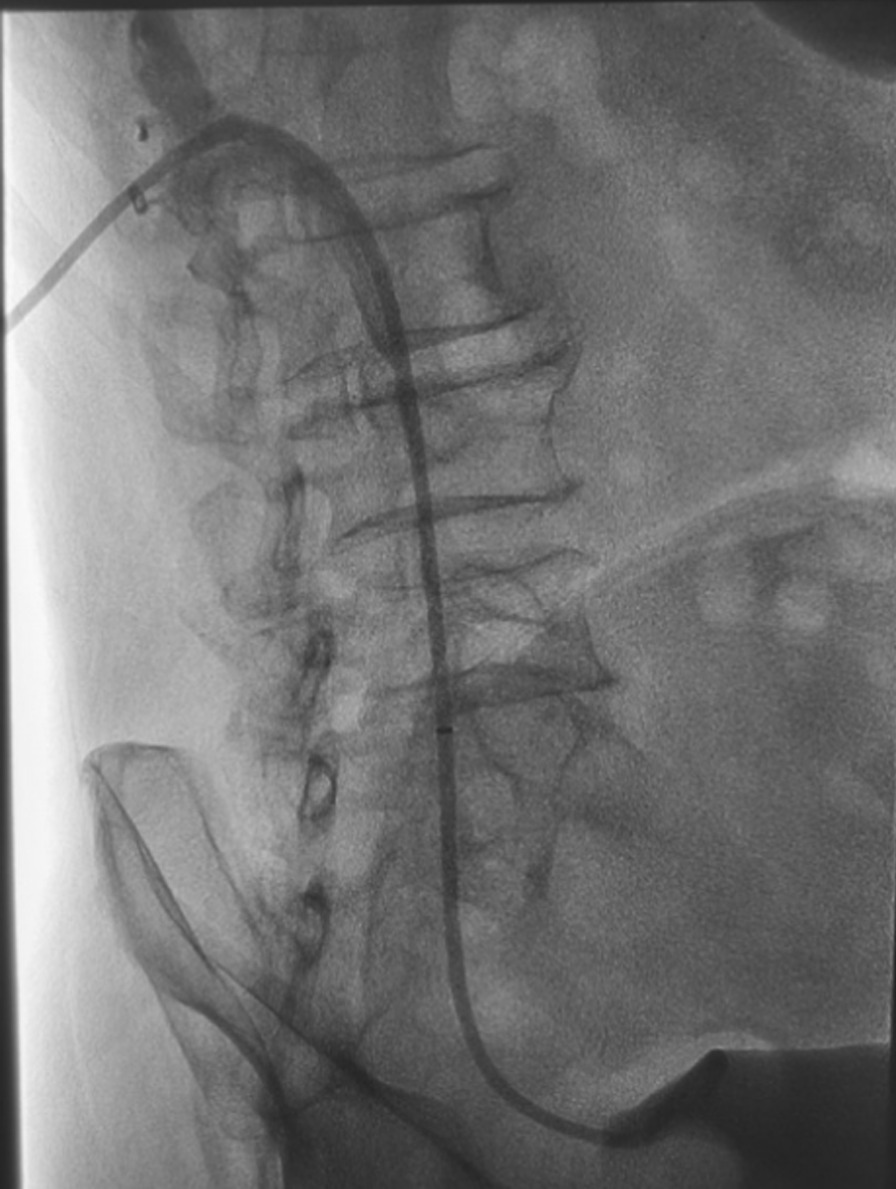
The internal and external drainage tube was implanted under fluoroscopy, with the distal end of the tube located in the bladder and the side holes of the tube located in the renal pelvis

**Fig. 3:**
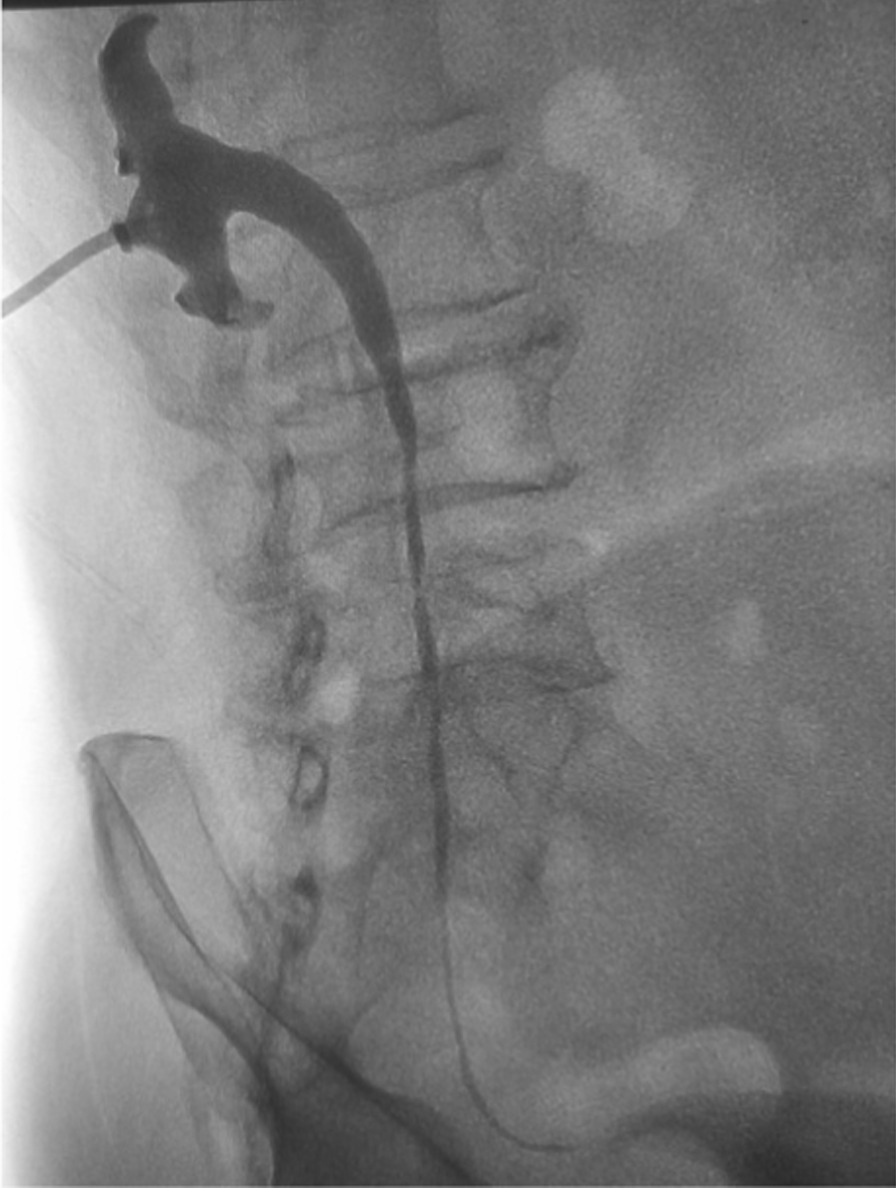
The end of the drainage tube is pulled into the renal pelvis. The contrast medium smoothly enters the bladder cavity through the ureter by drainage tube radiography

Endoscopic retrograde ureteral dilatation with double-J stent implantation and renal puncture balloon dilatation with internal and external drainage tube implantation are both standard treatment methods in our center, and which treatment method is selected is evaluated according to the imaging data of patients before treatment. In this group of patients, we chose the latter treatment modality. After local anesthesia, Chiba needle was punctured into the renal calyces of the middle group under the guidance of ultrasound. 6F catheter sheath was introduced under Digital subtraction angiography (DSA), and 5F multipurpose catheter was introduced through the catheter sheath. Contrast agent was injected to show that the renal pelvis and calyces were dilated, the lower ureter was narrow, and the proximal ureter was significantly dilated. During the operation, we selected a balloon with a diameter of 6 mm or 8 mm by referring to the diameter of the ureter in the normal area of the patient. The balloon catheter was used to dilate the ureteral stricture (Fig. 1). The inflation time of the balloon should not exceed 5 min each time; the balloon can be inflated repeatedly, but at an interval of 3–5 min. Prepare the 10.2F internal and external drainage tube, and open the appropriate side hole in the proximal part. The guide wire was retained and the sheath was pulled out. A 10.2F internal and external drainage tube was inserted through the guide wire, and the distal end of the tube was looped into the bladder cavity. The proximal side hole of the catheter is located in the renal pelvis, and the end of the catheter is connected with a drainage bag (Fig. 2). The drained urine was routinely tested and cultured, and targeted treatment was performed based on the results. After the hematuria disappears, the connecting port outside the body of the internal and external drainage tubes is closed with a heparin cap and no longer connected to the drainage bag. The drainage tube was washed with gentamicin saline once a month. Renal function and imaging examination were performed every two months to evaluate whether the drainage was sufficient. According to previous experience, the incidence of ureteral restenosis is very high after removal of the drainage tube within 6 months, so we routinely keep the drainage tube for 6 months. Six months later, the end of the internal and external drainage tube was pulled out from the bladder cavity to the renal pelvis under fluoroscopy, and the contrast agent was injected through the tube (Fig. 3).

The criteria for tube removal: Imaging examination showed that there was no ureteropelvic dilatation. Renal function tests revealed a significant decrease in urea nitrogen and creatinine levels. The distal end of the drainage tube was moved from the bladder to the renal pelvis before tube removal, and contrast agent was injected through the external orifice of the tube, which showed that the contrast agent smoothly entered the bladder from the renal pelvis through the ureter. If the above criteria are met, remove the tube. If not met, the end of the drainage tube is repositioned into the bladder with a guide wire. Repeat the above operations after three months.

### Follow up

**Fig. 4:**
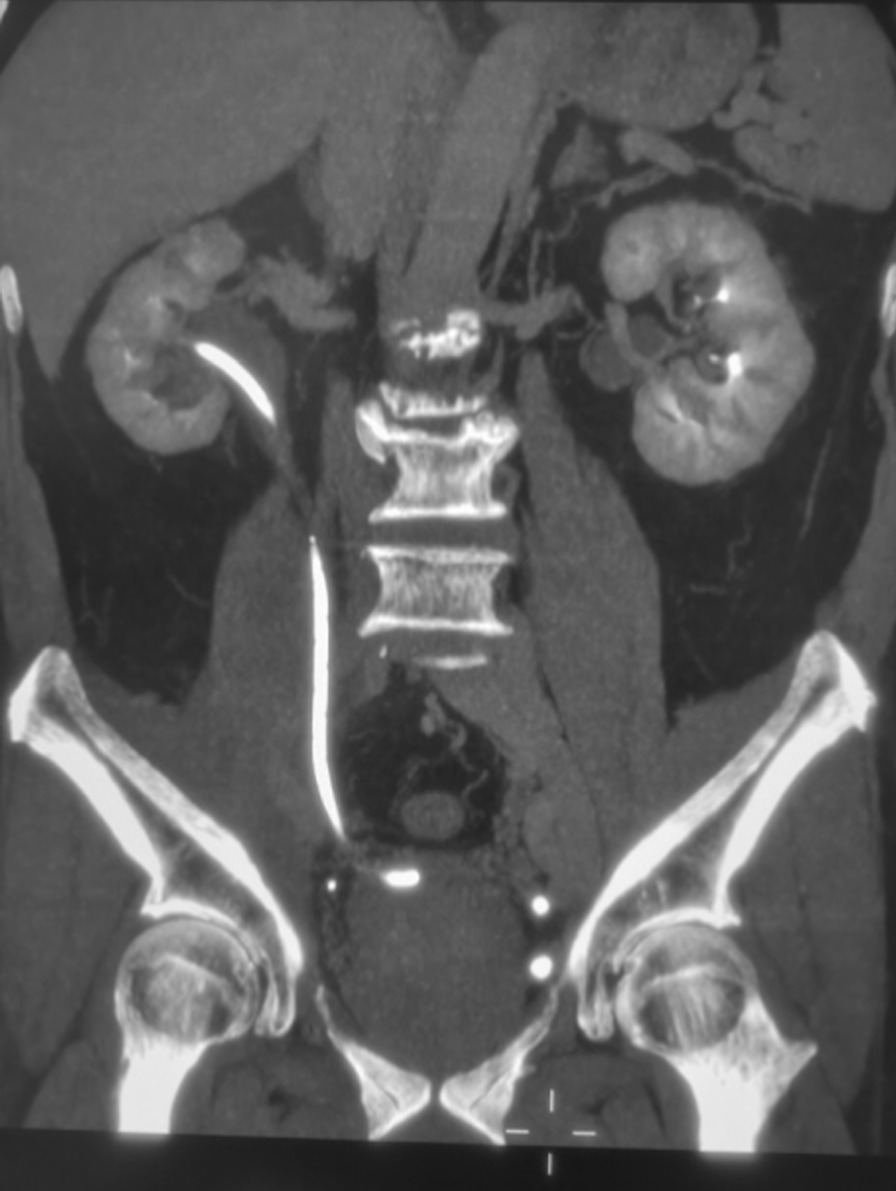
Recheck CT to confirm that the position of drainage tube is satisfactory

After the internal and external drainage tubes were removed, B-ultrasound, enhanced CTU or IVP of urinary system were reexamined every six months (Fig. 4). The overall success rate is defined as the patency rate at 36–60 months of follow-up.

### Statistical analysis

All analyses were performed using SPSS software (Version 24.0, IBM, Armonk, New York). The measurement data were expressed as mean ± standard deviation. The counting data are expressed as percentages. Paired T test was used for renal function before operation and tube removal, Cox regression analysis was used for multivariate analysis, *P* < 0.05 showed that the difference was statistically significant.

## Results

There were 25 male patients and 17 female patients. The average age was 52.9 ± 11.6 years (range, 27–73 years). According to age, there were 6 young (< 40 years old), 23 middle-aged (40–60 years old) and 13 elderly (> 60 years old) (Tables 1, 2). There were 6 cases of mild hydronephrosis, 15 cases of moderate hydronephrosis and 21 cases of severe hydronephrosis. The length of ureteral stricture was 0.2–2.1 cm, with an average of 1.1 ± 0.5 cm. The length of ureteral stricture was graded according to the following criteria. Mild stenosis: stenosis length < 0.8 cm (18 cases), moderate stenosis: stenosis length 0.8–1.5 cm (16 cases), severe stenosis: stenosis length > 1.5 cm (8 cases). There were 7 cases (16.7%) with congenital ureteral stricture, 12 cases (28.6%) with inflammation, 15 cases (35.7%) with ureteral stricture after lithotomy or lithotripsy, and 8 cases (19.0%) with ureteral stricture after pelvic or abdominal surgery. Preoperative urea nitrogen was 3.9–12.9 mmol/L, with an average of 9.2 ± 2.3 mmol/L. Preoperative creatinine was 54.5–339.5 umol / L, with an average of 175.8 ± 82.8 umol / L (Tables 1, 2).

**Table 1:** General information of patients

	Frequency	Percent (%)
*General information*		
*Gender*		
Female	17	40.5
Male	25	59.5
*Age group*		
Youth	6	14.3
Middle age	23	54.8
Old age	13	30.9
*Pathogeny*		
Congenital stenosis	7	16.7
inflammation	12	28.6
Urinary calculi related diseases	15	35.7
Iatrogenic	8	19.0
*Balloon diameter *(*mm*)		
6 mm	9	21.4
8 mm	33	78.6
*Degree of stenosis*		
Light	18	42.9
Moderate	16	38.1
Severe	8	19.0
*Degree of hydronephrosis*		
Light	6	14.3
Moderate	15	35.7
Severe	21	50.0
*Indwelling time of drainage tube (month)*		
6	33	78.6
9	9	21.4

**Table 2:** Descriptive statistics of patients' data

	Minimum	Maximum	Mean	SD
Age (y.o.)	27	73	52.93	11.564
Preoperative BUN (mmol/L)	3.90	12.90	9.2357	2.28065
Preoperative Cr (umol/L)	54.50	339.50	175.7619	82.80711
Indwelling time of drainage tube (month)	6	9	6.64	1.246
Hematuria disappearance time (day)	1	4	1.86	.872
BUN before tube removal (mmol/L)	3.80	9.10	6.2774	1.36499
Cr before tube removal (umol/L)	45.20	189.60	84.3786	27.25897
Follow-up time (month)	12	60	42.57	16.805
Narrow length (cm)	.20	2.10	1.0738	.53468

All 42 patients completed the interventional operation, and the technical success rate was 100%. The mean follow-up time was 42.6 ± 16.8 months (range, 12–60 months). In all 42 patients, all the internal and external drainage tubes were successfully removed according to our criteria for tube removal. There were 9 patients with 6 mm diameter balloon and 33 patients with 8 mm diameter balloon during the operation. After the internal and external drainage tube was implanted, no complications such as ureteral perforation and rupture occurred. The clearance time of hematuria in the drainage bag was 1–4 days, with an average of 1.9 ± 0.9 days. The internal and external drainage tubes indwelling time: 6 months in 33 cases, 9 months in 9 cases (Table 1). Urea nitrogen was 6.3 ± 1.4 mmol/L, and the creatinine was 84.4 ± 27.3umol/L when the internal and external drainage tubes were removed (Table 2). There were significant differences in the levels of urea nitrogen and creatinine before operation and during tube removal (*P* < 0.05) (Table 3). There were 5 patients with bladder irritation after drainage tube implantation, who improved with oral antibiotic therapy.

**Table 3:** Comparison of renal function before operation and before tube removal (BUN;Cr)

	Mean	N	SD	SE mean	
*Paired samples statistics*					
Pair 1	Preoperative BUN	9.2357	42	2.28065	.35191
	BUN before tube removal	6.2774	42	1.36499	.21062
Pair 2	Preoperative Cr	175.7619	42	82.80711	12.77741
	Cr before tube removal	84.3786	42	27.25897	4.20615

	*P* value
*Paired samples T test*	
*Pair 1*	
Preoperative BUN	.000
BUN before tube removal	
*Pair 2*	
Preoperative Cr	.000
Cr before tube removal	

There were 3 cases of ureteral restenosis in 12 months, 4 cases in 18 months, 2 cases in 24 months, 1 case in 30 months and 1 case in 36 months. The ureteral patency rate was 100% at 6 months, 93% at 12 months, 83% at 18 months, 79% at 24 months, 76% at 30 months and 73% at 36–60 months (Fig. 7). The overall success rate was 73%.

**Fig. 5:**
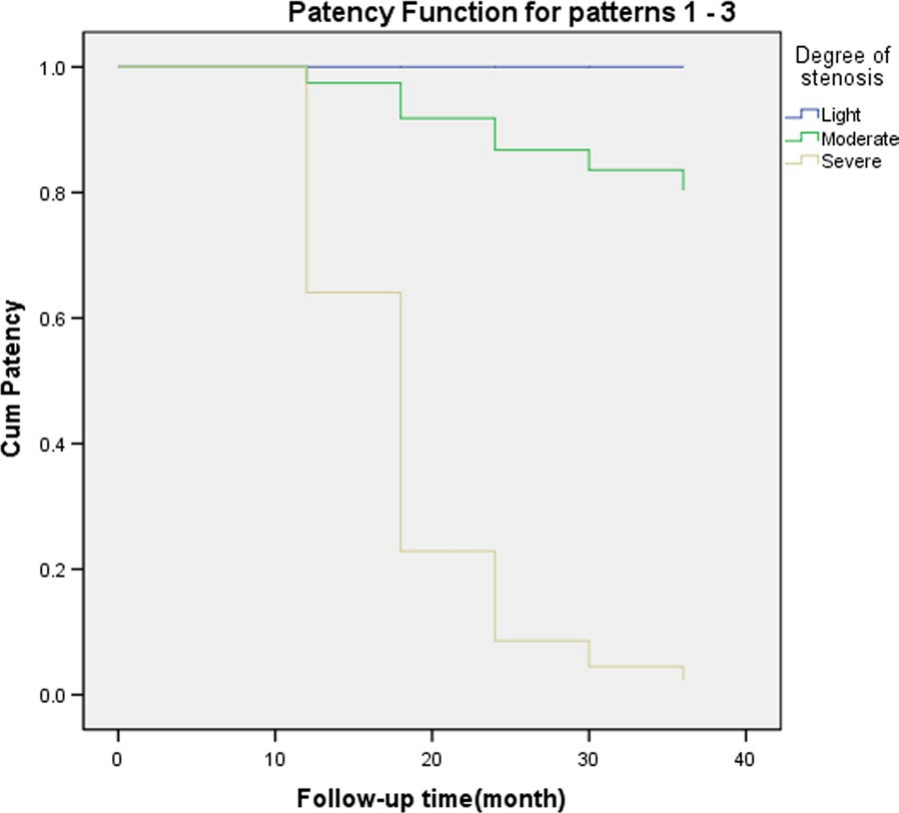
Patency rate of the drainage tube during follow-up in patients with different degrees of stenosis

**Fig. 6:**
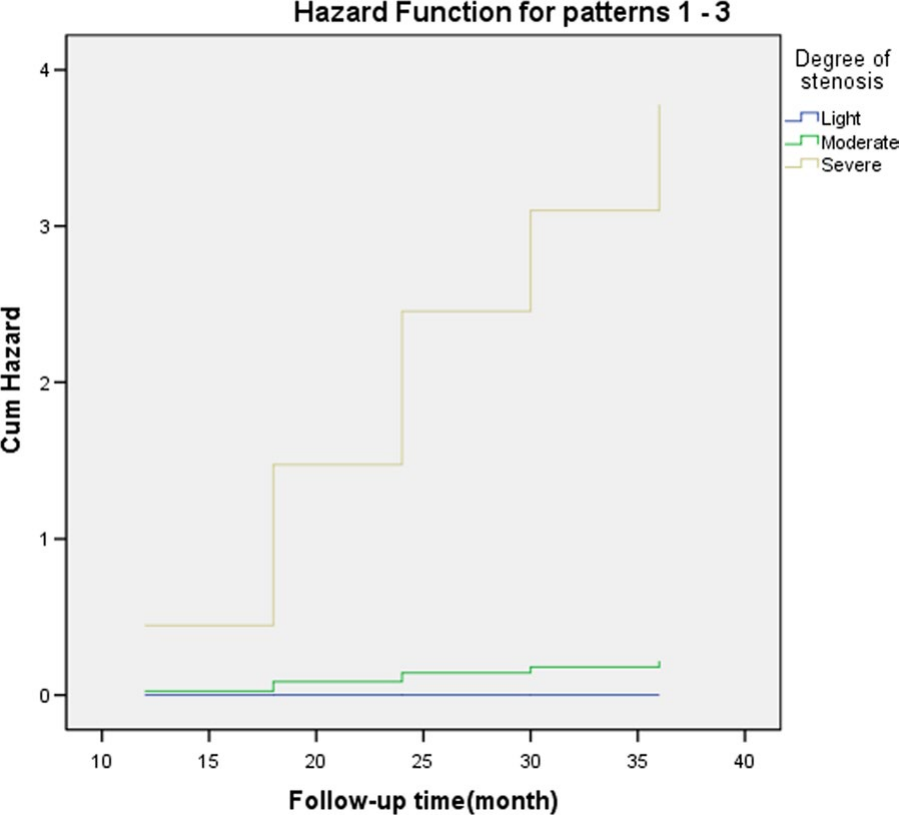
Cox regression analysis showed that the degree of stenosis was a risk factor for patency rate (*P* < 0.05)

Multivariate Cox regression analysis showed that the degree of stenosis was a risk factor for patency rate (*P* < 0.05) (Table 4, Figs. 5 and 6).

**Table 4:** Cox Regression Analysis of risk factors for restenosis

	B	SE	Wald	*df*	Sig	Exp (B)	95.0% CI for Exp (B)	
							Lower	Upper
*Variables in the equation*								
Gender	.157	1.024	.024	1	.878	1.170	.157	8.703
Age group			3.030	2	.220			
Age group (1)	.050	147.396	.000	1	1.000	1.052	.000	3.057E + 125
Age group (2)	2.320	147.404	.000	1	.987	10.179	.000	3.007E + 126
Pathogeny			6.354	3	.096			
Pathogeny (1)	−3.205	141.693	.001	1	.982	.041	.000	1.649E + 119
Pathogeny (2)	−.031	141.687	.000	1	1.000	.969	.000	3.895E + 120
Pathogeny (3)	−.779	141.687	.000	1	.996	.459	.000	1.844E + 120
Balloon diameter	.057	1.408	.002	1	.968	1.058	.067	16.700
Degree of stenosis			**8.412**	**2**	**.015**			
Degree of stenosis (1)	8.434	37.020	.052	1	.820	4602.698	.000	149517329022852150000000000000000000.000
Degree of stenosis (2)	11.284	37.025	.093	1	.761	79,558.091	.000	2.610E + 036

Cox regression analysis (bold) showed that the degree of stenosis was a risk factor for patency rate (*P* < 0.05)

## Discussion

Commonly used treatments for ureteral strictures include open surgery and minimally invasive treatment. The methods of minimally invasive treatment include ureteroscopic treatment and interventional treatment. Ureteroscopic treatment includes transurethral cold knife ureterotomy, holmium laser ureterotomy [8, 9], rigid ureteroscopic dilatation, balloon dilatation, ureteral stent implantation, etc. Esteban Emiliani et al. [10] reported that endoureterotomy and endopyelotomy should be considered a reasonable treatment option in selected patients of ureteral strictures. Ureteroscopy is generally retrograde to the ureter through the bladder. For lower ureteral stricture, due to the stricture of the ureteral opening or the failure of the guide wire to travel far in the ureter, the operation can not be completed retrogradely [2, 11, 12]. Therefore, for lower ureteral stenosis, antegrade interventional therapy has certain advantages. Antegrade interventional therapy includes balloon dilatation and ureteral stent implantation. Li et al. [13] reported 78 cases of lower ureteral stenosis, using percutaneous nephrostomy combined with balloon dilatation, the effective rate was 92%. In this group of 42 patients, the technical success rate was 100%, which was consistent with that reported in the literature.

Banner et al. [14] first reported the use of balloon dilatation in the treatment of ureteral stricture in 1983. A large number of studies have confirmed that balloon dilatation has a significant effect in the treatment of ureteral stenosis [15, 16], and has become one of the main treatment for benign ureteral stenosis [1]. Wai Loon Yam et al. [17] found that balloon dilatation of benign ureteric stricture is a feasible option, its effect can be long-lasting in selected patients. Jianhua Li et al.[13] demonstrated that percutaneous renal access anterograde flexible ureteroscope plus retrograde balloon dilatation is safe, effective and mini-invasive. Yam et al. [18] reported that balloon dilatation is safe and effective in the treatment of ureteral stricture, and the effective duration is long. The main complications were mucosal injury, ureteral perforation, hematuria and lumbar discomfort. The most common postoperative complication in this group of patients was hematuria, which lasted for 1–4 days. No serious complications such as ureteral rupture occurred. Kuntz NJ et al. retrospectively analyzed 151 cases of ureteral stricture treated by balloon dilatation, the success rate of operation was 95%, and the incidence of intraoperative complications was 5% [19]. Referring to the depth of ureteroscopic cold knife incision for the whole layer of the ureter, directly to the adipose tissue around the ureter [20], we believe that the full expansion of the ureter is helpful to improve the therapeutic effect. In this group of 42 patients, our selected balloon diameter was 6–8 mm, and no complications such as ureteral rupture occurred.

In 1967, Zimskind first implanted the stent into the ureter to relieve ureteral obstruction. In 1987, double-J stent was used for the first time. The commonly used ureteral stents are double-J stent [21], metal stent [22, 23], new anti biological peptide stent, etc. According to different conditions, it is very important to choose the appropriate stent [24]. For benign ureteral stricture, double-J stent is the most commonly used. Metal stents are mostly used for malignant ureteral strictures [25]. Ureteral stents can be used alone or in combination after balloon dilatation or endoscopic treatment [26]. Studies have shown that double-J stent after balloon dilatation can prevent ureteral rebound and scar contraction, help to drain urine and restore renal function. Hua-liang Yu et al. [27] found that treatment of benign ureteral stricture by double J stents using high-pressure balloon angioplasty produces a better therapeutic effect.

The commonly used drainage methods include internal drainage, external drainage and internal – -external drainage [28, 29]. External drainage refers to the implantation of nephrostomy tube after renal puncture. Internal drainage is accomplished by placement of a ureteral stent [30]. Internal–external drainage is performed by simultaneous ureteral stent implantation and external drainage tube implantation after renal puncture [31]. There are many benefits to preserving the external drainage tube when implanting a double-J stent by renal puncture. Because hematuria is often present shortly after renal puncture, blood clots may block the double-J stent. In the stage of hematuria, opening the external drainage tube can avoid double-J stent blockage. If it is necessary to replace the double-J stent, it can also be operated through the access of the external drainage tube. There are few reports about indwelling internal and external drainage tube after balloon dilatation. In this group, 10.2F internal and external drainage tube is used. Using this method, the patient does not need an external drainage bag, and the urine can flow into the bladder through the tube. The tube can be flushed regularly through the opening left outside the body to prevent blockage. The opening retained outside the body can be used as a path for removing or replacing the tube, which is directly operated and very convenient. Different from the double-J stent, the removal or replacement must use cystoscopy or catcher, which is complex and more painful for patients. The tube can be pulled out directly, and the success rate of tube removal is higher than that of double J stent. According to the research report, the success rate of percutaneous antegrade removal of double J stent is 93–97% [32, 33]. Cheng-Shi Chen [34] reported the success rate of retrograde removal of double-J stent was 97.44% (304/312). The success rate of tube removal in this study was 100%, which was higher than the success rate of double-J stent removal reported in the literature.

**Fig. 7:**
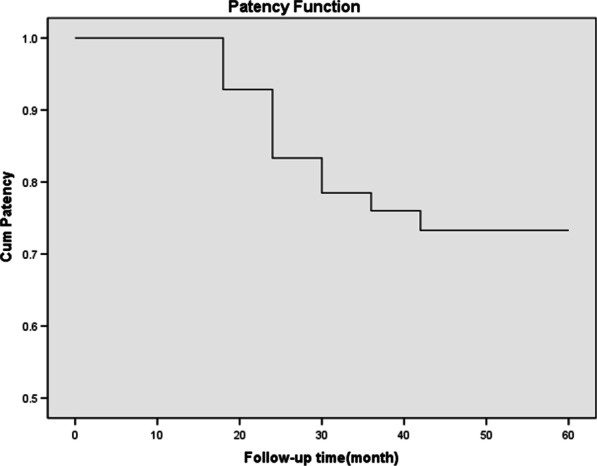
The ureteral patency rate was 100% at 6 months, 93% at 12 months, 83% at 18 months, 79% at 24 months, 76% at 30 months and 73% at 36–60 months

It has been reported that in the treatment of ureteral strictures, the indwelling time of double-J stent is about 6 weeks–3 months [35–37]. If it is necessary to continue indwelling, the double-J stent must be replaced [38]. In this study, we retained the internal and external drainage tubes for 6 months before reexamination. According to the previous experience at our center, the risk of restenosis is high after removal of the tube within 6 months. Although the usual indwelling time of double-J stent is 6 weeks to 3 months, some scholars have put forward different opinions. The study by Li et al. suggests that the indwelling time is related to the efficacy, and the recommended indwelling time is 6 months [39]. The indwelling time of internal and external drainage tube is longer due to regular flushing. The longest indwelling time in this group was 9 months, no tube blockage occurred. All drainage tubes were successfully removed on schedule. In this group, restenosis mainly occurred within 36 months, especially within 18 months, which indicates that ureteral restenosis is easy to occur in the process of ureteral repair (Fig. 7) Therefore, we suggest balloon dilatation combined with internal and external drainage tube implantation to prevent ureteral restenosis. After 36 months of follow-up, there was no ureteral stricture in this group, This indicates a low incidence of mid- to long-term restenosis after stable repair of the ureter.

In this group, the 6-month ureteral patency rate was 100%, the 12-month ureteral patency rate was 93%, the 18 month ureteral patency rate was 83%, the 24 month ureteral patency rate was 79%, the 30 month ureteral patency rate was 76%, and the 36–60 month ureteral patency rate was 73% (Fig. 7). The overall success rate was 73%. This is consistent with the success rate of 76.5% reported by Han [9] for endoureterotomy. There are many prognostic factors affecting the efficacy, such as the cause of stenosis [40], the length of stenosis [41], the time of stenosis [42], stent indwelling time [43] and the location of stenosis. According to the prospective study of Byun SS et al. [44], the length and etiology of ureteral stenosis are the factors influencing the effect of balloon dilatation. It has been reported that the successful rate of balloon dilatation is high when the stenosis length is less than 2 cm. The success rate of ureteral dilatation is high when the stenosis time is less than 3–6 months [15]. Cox regression analysis showed that the degree of ureteral stricture was a risk factor for restenosis, which is consistent with the results of other studies. However, the sample size of this group is relatively small, and it is a single center retrospective analysis. In the later stage, we can design a multi center, prospective, randomized controlled study to obtain more reliable research results.

## Conclusion

Balloon dilatation combined with internal and external drainage tube implantation in the treatment of benign lower ureteral stricture is a safe and reliable treatment strategy, with satisfactory short-term and long-term effects.

## Data Availability

The datasets used and analysed during the current study are available from the corresponding author on reasonable request.
